# Clinical Outcomes of Bone‐Level and Tissue‐Level Short Implants Placed in Posterior Maxilla: A Case–Control Study

**DOI:** 10.1111/cid.13428

**Published:** 2024-12-15

**Authors:** Teresa Lombardi, Antonio Rapani, Fatima Ezeddine, Giulia Perazzolo, Roberto Di Lenarda, Stefano Sivolella, Claudio Stacchi

**Affiliations:** ^1^ Department of Health Sciences “Magna Græcia” University Catanzaro Italy; ^2^ Department of Medical, Surgical and Health Sciences University of Trieste Trieste Italy; ^3^ Department of Neurosciences University of Padova Padova Italy

**Keywords:** abutments, atrophic maxilla, crestal bone resorption, implant survival

## Abstract

**Introduction:**

Short implants are today a reliable, minimally invasive option for the rehabilitation of the posterior maxilla. However, maintaining marginal bone stability remains a crucial factor for long‐term success, particularly in the case of short implants. The present multicenter prospective case–control study aimed to compare the clinical outcomes of bone‐level and tissue‐level short implants in the posterior maxilla, focusing on implant survival and peri‐implant marginal bone stability over 1 year of function.

**Methods:**

Fifty‐nine patients who met specific inclusion criteria were enrolled and treated by three clinical centers with a total of 74 short implants, either bone‐level (7 mm in length, placed 1 mm sub‐crestally) or tissue‐level (5 or 6.5 mm in length). The primary outcome was physiological bone remodeling (PBR) measured via radiographs at baseline (T0), prosthesis delivery (T1), and 12 months post‐loading (T2). Statistical analysis was performed to evaluate differences in PBR between groups, with multivariate analysis assessing the influence of various patient and site‐specific factors.

**Results:**

The final analysis included 58 patients who were treated with a total of 71 short implants, comprising 36 tissue‐level and 35 bone‐level implants (one patient dropped out as he did not attend follow‐up visits on time). All implants were rehabilitated with fixed, screwed prosthetics after 5 months, with no recorded complications up to 1 year of loading. Stability was similar between the two implant types at T0 and T1, with no significant differences in insertion torque and implant stability quotient (ISQ). Multivariate analysis revealed a significant positive correlation between insertion torque and ISQ at T0, as well as with bicortical engagement of the implant apex with the sinus floor. Tissue‐level implants demonstrated significantly lower peri‐implant bone remodeling (PBR) compared to bone‐level implants at both T1 (0.11 ± 0.27 mm vs. 0.34 ± 0.35 mm, *p* = 0.004) and T2 (0.30 ± 0.23 mm vs. 0.55 ± 0.42 mm, *p* = 0.003). Multivariate analysis showed a significant positive correlation between PBR (T0–T1) and thin vertical mucosal thickness (≤ 2 mm) at T0 in both tissue‐level and bone‐level implants. Additionally, PBR (T1–T2) in both groups significantly correlated with the use of short prosthetic abutments (≤ 2 mm) and, only in bone‐level implants, with crown emergence angles > 30°.

**Conclusion:**

Both tissue‐level and bone‐level short implants are effective options for implant‐supported rehabilitation in the posterior maxilla. Tissue‐level short implants offer superior marginal bone stability compared to bone‐level implants placed subcrestally, suggesting their favorable use in clinical practice.

## 1 Introduction

Implant‐supported rehabilitation of the edentulous posterior maxilla may present challenging clinical difficulties due to the poor bone quality of the area, often associated with limited available bone height after post‐extractive alveolar bone resorption and sinus pneumatization [1, 2]. Various surgical strategies are available to overcome these issues, including short and tilted implants, maxillary sinus floor elevation, vertical and horizontal augmentation of the atrophic bone crest, and pterygoid or zygomatic implants [3, 4, 5, 6, 7, 8, 9]. This broad range of therapeutic options should be thoroughly evaluated by the clinician, who should weigh the advantages and disadvantages of the various approaches in each individual patient.

Short implants are currently considered an interesting minimally invasive option also in the posterior maxilla [10]. Numerous recent clinical studies, systematic reviews, and meta‐analyses reported comparable or even more favorable clinical outcomes for implants < 8 mm when compared to standard‐length implants associated with sinus augmentation procedures [3, 4, 11, 12, 13, 14]. Moreover, short implants reduce patient morbidity, time of therapy, and economic costs [4, 10].

Stability of peri‐implant marginal bone levels, which has always been considered among the most important parameters to evaluate implant success [15], is even more crucial when implant length is limited. Loss of crestal bone around short implants not only exposes the implant to a higher risk of peri‐implant pathologies but may also represent a significant reduction of total implant bony support [16]. In particular, adequate control of physiological bone remodeling is fundamental to prevent further peri‐implant bone loss and decrease the risk of peri‐implant pathologies over time [17, 18]. However, it should be emphasized that this concept is mainly valid when using implants with treated surfaces. In fact, there are various studies in the literature that demonstrate how the physiological bone remodeling is not a predictor of further bone loss and/or implant failure when using machined implants [19, 20, 21].

Among the various factors potentially influencing physiological bone remodeling (insufficient bone envelope, traumatic surgery, excessive cortical compression during implant insertion, supracrestal tissue adhesion, prosthetic abutment height, number of abutment connections/disconnections, crown–implant ratio, and restoration emergence profile) [22, 23, 24, 25, 26, 27, 28], the microbial colonization of the implant‐abutment micro‐gap plays a paramount role [29, 30]. Bacterial harboring into the micro‐gap induces local inflammation resulting in the formation of an area of infiltrated connective tissue that three‐dimensionally surrounds the implant‐abutment area, causing bone resorption [31].

Two main strategies have been developed to overcome this issue: improvement of the quality of implant‐abutment connections and the introduction of horizontal implant‐abutment mismatch (platform‐switching) to move micro‐gap location far from the bone crest. Internal and, in particular, conical connections allow a considerable reduction of micro‐gap dimensions when compared to flat‐to‐flat configuration (5–10 vs. 30–50 μm), limiting at the same time unwanted micro‐movements at the connection level under loading conditions [32]. Platform‐switching has also demonstrated to be an efficient strategy to limit peri‐implant bone resorption, provided that the distance between the micro‐gap and the bone crest is ≥ 0.4 mm [33].

Tissue‐level implants, in which the implant‐abutment junction is coronally displaced far from the bone crest, showed long‐term, extremely positive outcomes in terms of marginal bone preservation and connection stability [34]. However, a broader use in the clinical practice of tissue‐level implants has been hampered by some intrinsic limitations: the main drawback of this implant configuration is related to possible esthetic issues in case of thin supracrestal tissue height and/or soft tissue recessions.

To the best of the authors' knowledge, no comparative studies are present in the literature on the clinical outcomes of bone‐level and tissue‐level short implants when used in the rehabilitation of the posterior maxilla. Therefore, the aim of the present multicenter prospective case–control study is to compare implant survival and marginal bone stability of bone‐level and tissue‐level short implants placed in the posterior maxilla and followed for 1 year after prosthetic loading.

## 2 Materials and Methods

### 2.1 Study Design

The present study was designed as a multicenter, prospective case–control study, conducted by three experienced operators (T.L., S.S., C.S.), who enrolled and treated patients from June 2020 to May 2023. The present investigation follows the Strengthening the Reporting of Observational Studies in Epidemiology (STROBE) guidelines. The study protocol adheres to the recommendations of the Fortaleza revision (2013) of the Helsinki Declaration for research on human subjects and was approved by the Comitato Etico Regione Calabria—Sezione Area Centro (n. 194/2020 dd. 18/06/2020). The study was recorded in a public registry of clinical studies (https://clinicaltrials.gov—no. NCT05975138). The three clinical centers participated in a calibration meeting prior to the beginning of the study to standardize operative procedures and data collection, with the aim of reaching acceptable inter‐operator consistency.

All patients were thoroughly informed about the study protocol, the therapeutic program with associated possible risks, and viable alternatives. Patients willing to participate in this study signed a written informed consent and authorized the use of their clinical and personal data for research purposes.

The present superiority trial tested the null hypothesis of no difference in peri‐implant marginal bone loss after 1 year of function around tissue‐level (test group) and bone‐level (control group) short implants inserted in the posterior maxilla against the alternative hypothesis of a difference.

### 2.2 Patient Selection

All partially edentulous patients needing an implant‐supported rehabilitation in pristine bone in the posterior maxilla were evaluated for potential inclusion in this case–control study.

General inclusion criteria were the following:

- age > 18 years;
- good general health conditions;
- no systemic diseases influencing osseous metabolism and/or wound healing;
- no regular medication intake;
- patient able to comply with the study protocol;
- signed informed consent.

Local inclusion criteria were the following:

VII edentulous alveolar ridge with a minimum of 6 mm width and 4.5–8.0 mm height between the alveolar crest and the maxillary sinus floor, with no concomitant or previous bone augmentation procedures;
VIII time from tooth loss/extraction > 6 months;
IX absence of any removable prosthesis in the planned treatment area;
X presence of opposing dentition.

Exclusion criteria were:

- general contraindications to implant surgery [35];
- glycated hemoglobin > 7.5%;
- present or past treatment with antiresorptive drugs;
- pregnancy or breastfeeding;
- full mouth plaque score (FMPS) > 25%;
- untreated periodontal disease;
- substance abuse
- insertion torque < 20 N cm.

All patients were motivated for oral hygiene and underwent professional deplaquing 1 week before surgery.

A total of 59 consecutive patients who met the study inclusion criteria were recruited from three clinical centers (T.L.: 19; S.S.: 15; C.S.: 25) and treated with the insertion of 74 short implants, consisting of 37 tissue‐level and 37 bone‐level implants. All implants were placed with a one‐stage protocol and were rehabilitated with fixed prosthetics 5 months after placement.

The demographic characteristics of these patients are presented in Table 1.

**TABLE 1:** Demographic characteristics of the treated patients.

	Total (*n* = 59)
Age	64.1 ± 10.9
Gender (male/female)	26 (44.1%)/33 (55.9%)
Smoking habits (yes/no)	12 (20.3%)/47 (79.7%)
History of periodontitis (yes/no)	18 (30.5%)/41 (69.5%)

### 2.3 Clinical Procedures

**FIGURE 1:**
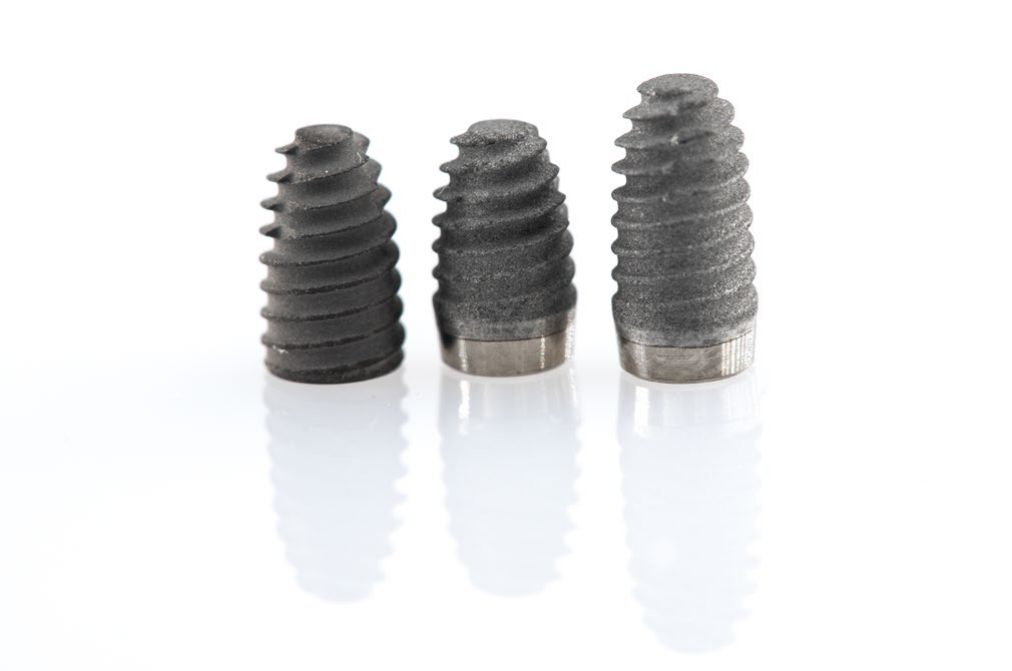
Short implants (M2, Zeros, Busan, South Korea) used in the present study. Left to right: 7‐mm‐length bone‐level implant; 5‐mm‐length tissue‐level implant; 6.5‐mm‐length tissue‐level implant.

After raising a minimally invasive full‐thickness buccal flap under local anesthesia (articaine 4% with epinephrine 1:100 000), supracrestal tissue height was measured with a periodontal probe (UNC 15, Hu‐Friedy, Chicago, USA) and categorized into thin (≤ 2 mm) or thick (> 2 mm) biotype [36]. After completing palatal flap elevation, the implant site was prepared with rotary instruments under abundant irrigation to allow placement of a 4‐mm‐diameter short implant (M2, Zeros, Busan, South Korea) with a conical hybrid connection. The most appropriate implant length (5, 6.5, or 7 mm) was chosen by the surgeon based on the available bone height below the sinus floor. A 5‐ and 6.5‐mm‐length fixtures are tissue‐level implants with a 2 mm high, machined, convergent trans‐epithelial neck, with the rest of the fixture presenting a moderately rough titanium surface. A 7‐mm‐length implant is a bone‐level implant fully treated with a moderately rough surface (Figure 1). Tissue‐level and bone‐level implants, excluding the neck area, present the same macro‐morphology and connection. As per the manufacturer's recommendations, tissue‐level implants were placed with the rough surface submerged in the bone and the neck at soft tissue level for transgingival healing, while bone‐level implants were positioned 1 mm subcrestally and immediately connected to a healing abutment.

Insertion torque (IT) was recorded by the surgical motor (Implantmed, W&H, Bürmoos, Austria) and implant stability quotient (ISQ) was measured after implant placement with a specific transducer (Smartpeg #27, Osstell, Göteborg, Sweden). Flaps were sutured in both groups for an unsubmerged healing with single sutures and/or the Sentineri technique using synthetic monofilament [37]. Antibiotic therapy (amoxicillin/clavulanate 1 g twice a day) for 5 days and nonsteroidal anti‐inflammatory drugs (ketoprofen 80 mg when needed) were prescribed. Sutures were removed after 12 days, and the course of healing was checked monthly.

After 4 months of healing, ISQ was measured and definitive impressions were taken. Screwed metal‐ceramic single crowns or three‐unit bridges were delivered, and patients entered into a 4‐month recall program to check and maintain oral hygiene standards.

### 2.4 Radiographic Measurements

**FIGURE 2:**
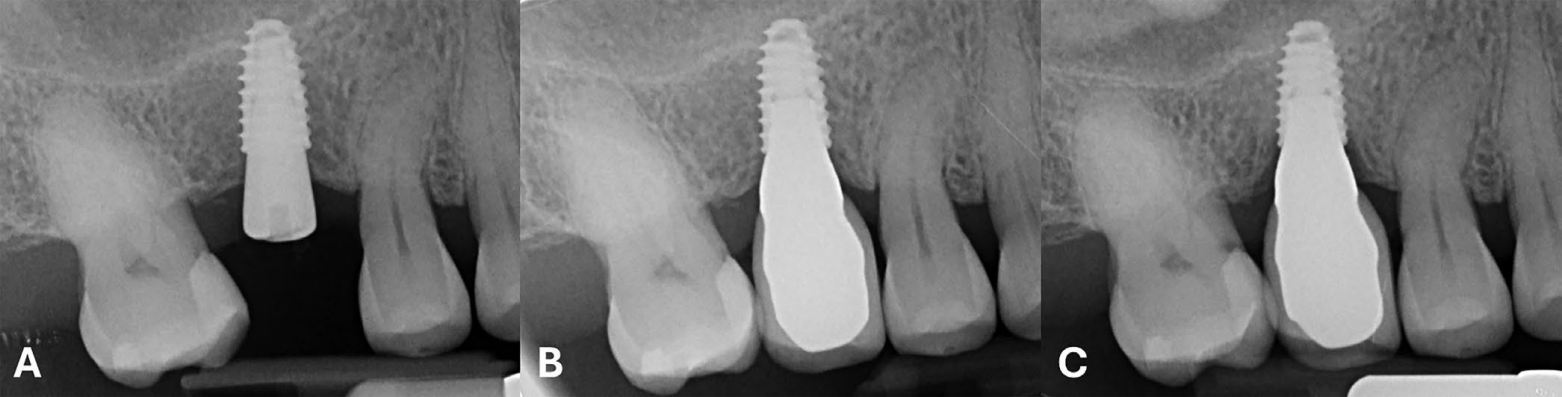
Bone‐level implant at implant positioning (A), crown delivery (B), and after 12 months of loading (C).

**FIGURE 3:**
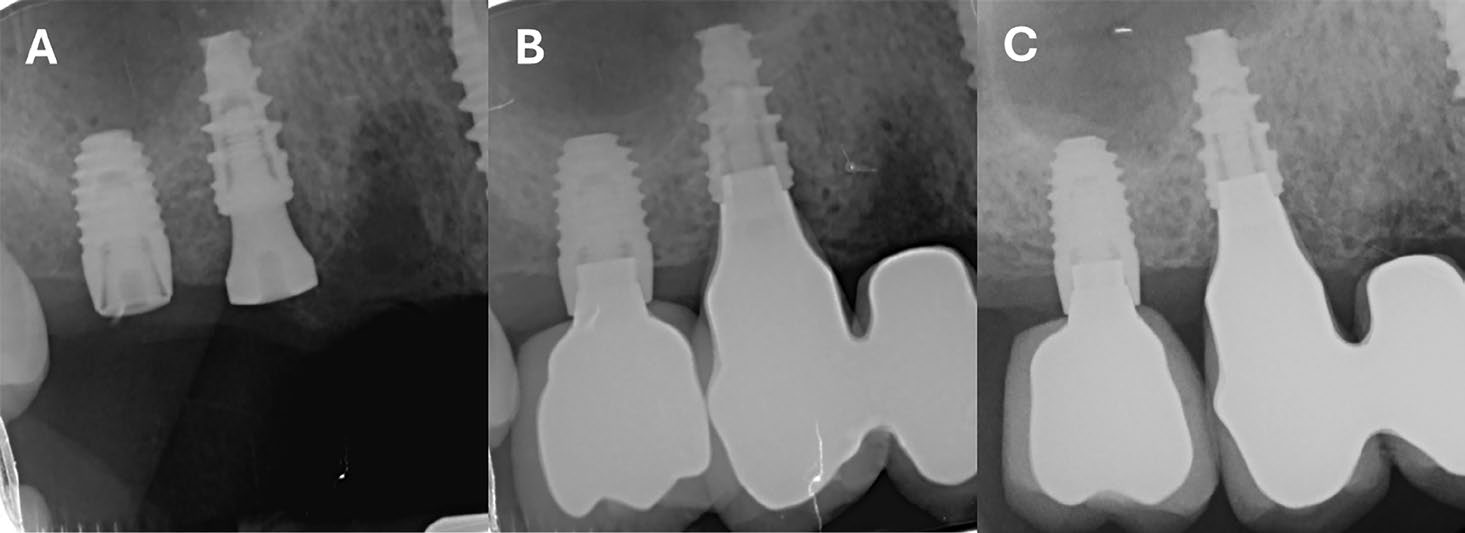
Tissue‐level implant at implant positioning (A), crown delivery (B), and after 12 months of loading (C).

Periapical radiographs were taken using a long‐cone paralleling technique with a customized Rinn‐type film holder at implant placement (T0), at prosthesis delivery (5 months after implant placement—T1), and after 12 months of function (T2) (Figures 2 and 3).

Variations in peri‐implant bone levels using T0 as a reference point were measured at each time interval on both the mesial and distal aspects of the implant. A positive value was assigned when bone level was more coronal than at T0, whereas a negative value was assigned when bone level was more apical than at T0.

All measurements were taken by two calibrated examiners (F.E. and G.P.) on a 28‐in. diagnostic monitor using measuring software (Image J 1.52a, National Institutes of Health, Bethesda, USA). Measurements were calibrated referring to the known implant diameter at platform level. Each measurement was taken three times at three different time points, as previously proposed by Gomez‐Roman and Launer [38]. Examiners were calibrated by assessing 12 radiographs, with a different assessor (S.S.) serving as a reference examiner. Intraclass correlation coefficients to assess intra‐observer and inter‐observer variability were 0.99 and 0.87, respectively, for linear measurements within ±0.1 mm.

Moreover, crown–implant ratio and interproximal crown emergence angles (mesial and distal) were measured on T1 radiographs using the same software. According to the Glossary of Prosthodontic Terms, emergence angle was defined as “the angle of an implant restoration's transitional contour as determined by the relation of the surface of the abutment to the long axis of the implant body” [39].

### 2.5 Predictor and Outcome Variables

Primary site‐specific predictor variables were the following: (i) crestal vertical mucosal thickness (thick > 2 mm vs. thin ≤ 2 mm) [40, 41]; (ii) implant insertion torque (> 50 vs. ≤ 50 N cm) [42, 43]; (iii) implant primary stability (≥ 60 vs. < 60 ISQ) [44]; (iv) bicortical engagement of the implant on the sinus floor (yes/no) [45]; (v) abutment height (long > 2 mm vs. short ≤ 2 mm) [25, 46]; (vi) crown–implant ratio [27]; (vii) crown emergence angle (> 30° vs. ≤ 30°) [28, 47]. The possible influence on the primary outcome of the following patient‐related variables was also analyzed: (i) age; (ii) gender; (iii) smoking habits (smoker vs. non smoker); (iv) history of periodontitis (yes/no).

Primary outcome (dependent variables):

- physiological bone remodeling (PBR) at T1 and T2.

Secondary outcomes:

- implant stability at T0 (peak insertion torque; ISQ);
- implant failure (implant mobility or implant removal due to progressive marginal bone loss);
- any biological or mechanical complication.

### 2.6 Sample Size Calculation and Statistical Power

The sample size of the present prospective study was calculated using a statistical software (Primer of Biostatistics 6.0, McGraw‐Hill Education, New York, USA), based on data reported in a previous publication on peri‐implant marginal bone level variations between bone‐level and tissue‐level implants after 1 year of function (−0.03 ± 0.74 and 0.26 ± 0.55 mm, respectively) [48]. A sample of 34 implants for each group was required to detect significant differences between the two groups (confidence level 5% with statistical power of 80%).

### 2.7 Statistical Analysis

Statistical analysis was performed by an independent assessor (A.R.) using IBM SPSS Statistics Version 26.0 software (IBM, Armonk, NY, USA). Descriptive statistics for continuous variables were presented as means and standard deviations for parametric data or as medians and interquartile ranges for non‐parametric data, while categorical variables were expressed as frequencies. The Shapiro–Wilk test was used to assess the distribution of the dataset at various time points, and the Levene test was applied to evaluate the equality of variances. Differences in continuous variables between the test and control groups were analyzed using a *t*‐test for parametric data and a two‐sample Wilcoxon rank‐sum test for non‐parametric data. Multiple linear regression analysis was performed to assess the impact of independent variables on the dependent variable. The “Enter” method was used to include all independent variables in the model. A *p*‐value of less than 0.05 was considered statistically significant.

## 3 Results

One patient, who received three implants (one 5 mm tissue‐level implant and two 7 mm bone‐level implants), was lost to follow‐up after prosthetic delivery, as he was no longer interested in participating in the study.

Therefore, the final analysis included 58 patients (25 men and 33 women, mean age 63.3 ± 11.3 years) who received 36 tissue‐level implants [5 mm length (*n* = 18) or 6.5 mm length (*n* = 18)] and 35 bone‐level implants (7 mm length), supporting 67 single crowns and 2 three‐unit bridges. Among patients included in the final analysis, 13 patients received two implants (one tissue‐level – 5.0 or 6.5– and one bone‐level – 7 mm) and 45 patients received a single implant.

The mean residual bone height between the alveolar crest and the floor of the maxillary sinus at the time of implant placement was 6.2 ± 2.3 mm. No complications or adverse events were recorded and after 1 year of prosthetic loading, all 71 implants were satisfactorily in function.

At T0, no significant differences in primary stability were observed between bone‐level and tissue‐level implants, either in terms of mean IT (bone‐level: 40.8 ± 11.3 N cm; tissue‐level: 41.9 ± 14.0 N cm; *p* = 0.56) or mean ISQ (bone‐level: 64.7 ± 10.3; tissue‐level: 66.8 ± 9.3; *p* = 0.42). Similarly, at T1, implant stability (ISQ) did not differ significantly between the two groups (bone‐level: 71.4 ± 6.5; tissue‐level: 69.5 ± 7.0; *p* = 0.10). Multivariate analysis revealed a significant positive correlation between IT and ISQ (T0). Additionally, IT showed a significant positive correlation with bicortical engagement of the implant apex in the sinus floor.

Mesial and distal PBR values at T1 and T2 were compared using a paired *t*‐test, which revealed no significant differences. Therefore, the average of mesial and distal PBR was used as the primary outcome in the subsequent analysis. Mean PBR (T0–T1) around tissue‐level implants (0.11 ± 0.27 mm) was significantly lower than around bone‐level implants (0.34 ± 0.35 mm) (*p* = 0.004). Similarly, mean PBR (T0‐T2) around tissue‐level implants (0.30 ± 0.23 mm) remained significantly lower than around bone‐level implants (0.55 ± 0.42 mm) (*p* = 0.003).

Multivariate analyses revealed a significant positive correlation between PBR (T0–T1) and vertical mucosal thickness ≤ 2 mm in both tissue‐level and bone‐level implants. However, no significant associations were found between PBR (T0–T1) and other possible influencing factors such as age, gender, smoking habits, history of periodontitis, peak insertion torque > 50 N cm, ISQ (T0) ≥ 60, or bicortical engagement of the implant apex in the cortical of the sinus floor (Tables 2 and 3).

**TABLE 2:** Multiple linear regression analysis to assess the impact of independent variables on the dependent variable PBR (T0–T1) of tissue‐level implants.

	Coefficient *B*	95% CI	*p*‐value
Gender			
Male	1		
Female	−0.001	[−0.182 to 0.180]	0.992
Age	0.003	[−0.005 to 0.011]	0.461
Smoking habits			
Yes	1		
No	0.066	[−0.137 to 0.270]	0.508
History of periodontitis			
Yes	1		
No	0.118	[−0.088 to 0.323]	0.250
Bicorticalism			
Yes	1		
No	−0.066	[−0.218 to 0.086]	0.380
Insertion torque			
> 50 N cm	1		
≤ 50 N cm	0.061	[−0.142 to 0.263]	0.543
ISQ (T0)			
≥ 60	1		
< 60	0.058	[−0.148 to 0.263]	0.570
Mucosal thickness			
Thick (> 2 mm)	1		
Thin (≤ 2 mm)	−0.410	[−0.578 to −0.241]	0.000 *

* Statistically significant.

Abbreviations: CI, confidence interval; ISQ, implant stability quotient.

**TABLE 3:** Multiple linear regression analysis to assess the impact of independent variables on the dependent variable PBR (T0–T1) of bone‐level implants.

	Coefficient *B*	95% CI	*p*‐value
Gender			
Male	1		
Female	−0.160	[−0.368 to 0.048]	0.127
Age	−0.008	[−0.017 to 0.001]	0.095
Smoking habits			
Yes	1		
No	0.241	[−0.013 to 0.496]	0.062
History of periodontitis			
Yes	1		
No	0.075	[−0.188 to 0.337]	0.564
Bicorticalism			
Yes	1		
No	0.008	[−0.207 to 0.223]	0.936
Insertion torque			
> 50 N cm	1		
≤ 50 N cm	−0.071	[−0.350 to 0.208]	0.605
ISQ (T0)			
≥ 60	1		
< 60	0.122	[−0.129 to 0.372]	0.327
Mucosal thickness			
Thick (> 2 mm)	1		
Thin (≤ 2 mm)	−0.480	[−0.685 to −0.275]	0.000 *

* Statistically significant.

Abbreviations: CI, confidence interval; ISQ, implant stability quotient.

In tissue‐level implants, PBR (T1–T2) showed a significant positive correlation with the presence of a short prosthetic abutment (≤ 2 mm). No significant associations were found with age, gender, smoking habits, history of periodontitis, crown–implant ratio, or emergence profile angle of the crown > 30° (Tables 4 and 5). In bone‐level implants, PBR (T1–T2) showed a significant positive correlation with both the presence of a short prosthetic abutment (≤ 2 mm) and the emergence profile angle of the crown > 30°. No significant associations were found with age, gender, smoking habits, history of periodontitis, or crown–implant ratio (Tables 6 and 7).

**TABLE 4:** Multiple linear regression analysis to assess the impact of independent variables on the dependent variable PBR (T1–T2) on the mesial aspect of tissue‐level implants.

	Coefficient *B*	95% CI	*p*‐value
Gender			
Male	1		
Female	−0.032	[−0.191 to 0.128]	0.687
Age	0.005	[−0.003 to 0.012]	0.232
Smoking habits			
Yes	1		
No	0.164	[−0.034 to 0.363]	0.101
History of periodontitis			
Yes	1		
No	−0.161	[−0.363 to 0.040]	0.112
Crown/implant ratio	−0.121	[−0.359 to 0.118]	0.310
Abutment height			
Long (> 2 mm)	1		
Short (≤ 2 mm)	−0.195	[−0.337 to −0.053]	0.009*
Mesial emergence angle			
≤ 30°	1		
> 30°	0.000	[−0.007 to 0.008]	0.941

* Statistically significant.

Abbreviation: CI, confidence interval.

**TABLE 5:** Multiple linear regression analysis to assess the impact of independent variables on the dependent variable PBR (T1–T2) on the distal aspect of tissue‐level implants.

	Coefficient *B*	95% CI	*p*‐value
Gender			
Male	1		
Female	0.015	[−0.200 to 0.229]	0.891
Age	0.009	[0.000 to 0.019]	0.058
Smoking habits			
Yes	1		
No	0.056	[−0.240 to 0.351]	0.703
History of periodontitis			
Yes	1		
No	−0.163	[−0.425 to 0.098]	0.211
Crown/implant ratio	0.002	[−0.347 to 0.351]	0.990
Abutment height			
Long (> 2 mm)	1		
Short (≤ 2 mm)	−0.253	[−0.470 to −0.035]	0.024 *
Distal emergence angle			
≤ 30°	1		
> 30°	0.010	[−0.006 to 0.025]	0.205

* Statistically significant.

Abbreviation: CI, confidence interval.

**TABLE 6:** Multiple linear regression analysis to assess the impact of independent variables on the dependent variable PBR (T1–T2) on the mesial aspect of bone‐level implants.

	Coefficient *B*	95% CI	*p*‐value
Gender			
Male	1		
Female	0.047	[−0.087 to 0.181]	0.477
Age	0.005	[−0.002 to 0.011]	0.166
Smoking habits			
Yes	1		
No	−0.053	[−0.207 to 0.101]	0.486
History of periodontitis			
Yes	1		
No	−0.040	[−0.221 to 0.142]	0.658
Crown/implant ratio	−0.012	[−0.192 to 0.169]	0.896
Abutment height			
Long (> 2 mm)	1		
Short (≤ 2 mm)	−0.302	[−0.576 to −0.132]	0.038*
Mesial emergence angle			
≤ 30°	1		
> 30°	0.014	[0.002 to 0.026]	0.019*

* Statistically significant.

Abbreviation: CI, confidence interval.

**TABLE 7:** Multiple linear regression analysis to assess the impact of independent variables on the dependent variable PBR (T1–T2) on the distal aspect of bone‐level implants.

	Coefficient *B*	95% CI	*p*‐value
Gender			
Male	1		
Female	−0.105	[−0.254 to 0.044]	0.161
Age	0.002	[−0.005 to 0.008]	0.589
Smoking habits			
Yes	1		
No	−0.031	[−0.199 to 0.137]	0.707
History of periodontitis			
Yes	1		
No	−0.155	[−0.338 to 0.029]	0.096
Crown/implant ratio	0.099	[−0.098 to 0.296]	0.313
Abutment height			
Long (> 2 mm)	1		
Short (≤ 2 mm)	−0.277	[−0.515 to −0.097]	0.045*
Distal emergence angle			
≤ 30°	1		
> 30°	0.015	[0.005 to 0.024]	0.003*

* Statistically significant.

Abbreviation: CI, confidence interval.

## 4 Discussion

The present multicenter, prospective case–control study aimed to evaluate and compare the clinical outcomes of tissue‐level and bone‐level short implants in the rehabilitation of the posterior maxilla, focusing on implant survival and peri‐implant marginal bone stability after 1 year of functional loading.

### 4.1 Implant Survival and Stability

The present study demonstrated high implant survival rates for both tissue‐level and bone‐level short implants, with all 71 implants remaining in function after 1 year. This finding aligns with existing literature that supports the use of short implants as a viable solution in cases of reduced bone height in the posterior maxilla. Recent randomized clinical trials with 10‐year follow‐up [49, 50], along with meta‐analyses [3, 4, 11, 12, 13, 14], indicate that short implants demonstrate equal or superior long‐term performance compared to standard implants combined with sinus floor elevation in terms of implant survival, marginal bone loss, and incidence of intra‐ and post‐operative complications.

Surface treatment and implant morphology are two essential factors for achieving predictable success with short implants. The use of moderately rough surfaces (*S*_a_ between 1 and 2 μm) allows for a significant increase in the bone–implant contact area, even with limited implant lengths, greatly impacting the success of the treatment. It is worth noting that studies conducted on short implants with minimally rough surfaces (*S*_a_ between 0.5 and 1 μm) placed in the posterior maxilla reported a failure rate of around 20% at 5 years [51, 52, 53, 54]. On the other hand, implant morphology must ensure predictable primary stability, even with a reduced fixture length in poor‐quality bone. Tapered design, increased diameter, and deep threads are features that help enhance the primary stability of an implant, all other conditions being equal [55]. The implants used in the present study present these characteristics, and the fixture morphology is identical in both the bone‐level and tissue‐level configurations. In fact, no significant differences were observed in terms of primary stability between bone‐level and tissue‐level implants, whether considering insertion torque or the implant stability quotient (ISQ). It should be emphasized that the multivariate analysis revealed a positive correlation between primary stability and bicortical engagement of the implant apex with the maxillary sinus floor. This finding is consistent with previous in vitro and clinical studies [45, 56] and should be carefully considered, especially in cases of poor‐quality bone and limited height. Bicortical anchorage helps to reduce tensile stress distribution at the crestal cortical level [57], significantly improving the mid‐term prognosis of short implants in the posterior maxilla [58]. Moreover, when performed following a strict protocol, it does not pose a risk to the health or homeostasis of the maxillary sinus [59].

### 4.2 Peri‐Implant Physiological Bone Remodeling

The primary outcome of the present study was the evaluation of physiological bone remodeling (PBR) up to 12 months of function, which is a critical indicator of implant success [15, 16, 17, 18, 60], particularly in short implants where bone support is inherently limited. Nevertheless, the literature presents contrasting findings regarding the predictive value of PBR for the long‐term success and survival of dental implants. Several studies suggest that early bone loss does not necessarily predict continued bone loss or implant failure [19, 20, 21]. Additionally, other studies have reported a negative correlation between cumulative marginal bone loss and implant failure rates over extended periods (25–35 years of function), raising questions about the diagnostic reliability of bone level changes for assessing implant longevity [61, 62]. It is important to emphasize, however, that all the studies mentioned above were conducted with machined implants presenting minimally rough surfaces (*S*_a_ between 0.5 and 1 μm), whereas the implants in the present study have moderately rough surfaces (*S*_a_ between 1 and 2 μm). Long‐term clinical studies and systematic reviews suggest that implant surface characteristics play a significant role in the progression and treatment outcomes of peri‐implantitis, with machined implants exhibiting the least bone loss and achieving the most favorable treatment outcomes [63, 64, 65].

The results of the present study indicated that bone‐level implants exhibited significantly greater mean PBR (T0–T1: 0.34 ± 0.35 mm; T0‐T2: 0.55 ± 0.42 mm) compared to tissue‐level implants (T0–T1: 0.11 ± 0.27 mm; T0‐T2: 0.30 ± 0.23 mm). This can be attributed to the fact that, in the present study, all implants underwent unsubmerged healing, leading to immediate microbial colonization of the microgap between the implant and healing abutment [66, 67]. The presence of a microgap at bone level, regardless of the surgical approach, consistently results in a certain degree of crestal bone loss. This is driven by a predictable biological principle: Ericsson et al. [31] demonstrated that microbial colonization of the microgap triggers an inflammatory reaction, causing peri‐implant bone resorption and the formation of an inflammatory cell infiltrate around the microgap. Hermann et al. [68] further showed that in all two‐piece implant configurations, the extent of crestal bone loss was significantly affected by the microgap position relative to the bone crest. Significant bone loss and inflammation were observed when the microgap was at the bone crest level, while significantly less bone resorption and peri‐implant inflammation occurred when the microgap was positioned 1 mm above the crest.

Multivariate analyses showed that peri‐implant bone remodeling (PBR) from T0 to T1 in both tissue‐level and bone‐level implants was significantly associated with thin vertical mucosal thickness (≤ 2 mm). This finding aligns with several studies on the formation of supracrestal tissue adhesion around implants. Berglundh and Lindhe [69] were the first to observe that when the ridge mucosa was thin (≤ 2 mm) before abutment connection, wound healing consistently involved bone resorption. When an implant is exposed to the oral cavity, the soft tissue forms a protective barrier against bacterial infiltration into the underlying alveolar bone [70]. This process, known as supracrestal tissue adhesion [24], takes about 8 weeks for soft tissue maturation and results in the formation of a biological seal with an average vertical dimension in humans of 2.7 ± 0.4 mm (range 2.1–3.2 mm) [71]. If the vertical dimension of the peri‐implant mucosa is insufficient to form an adequate supracrestal tissue adhesion, peri‐implant marginal bone resorption occurs during healing to create additional space. This phenomenon has been well documented in numerous clinical studies [40, 41, 72, 73, 74]. Additionally, it is important to note that the average supracrestal tissue height around tissue‐level implants in humans has been shown to be lower than that around bone‐level implants [75].

During the subsequent period (T1–T2, from prosthesis delivery to 12 months of functional loading), multivariate analyses revealed a significant correlation between peri‐implant bone remodeling (PBR) around both tissue‐level and bone‐level implants and the use of short prosthetic abutments (≤ 2 mm). This is in accordance with numerous clinical studies and meta‐analyses reaching the same conclusions [25, 46, 76, 77, 78, 79, 80]. Possible explanations involve the encroachment of the crown into the space occupied by newly‐formed supracrestal tissue and issues related to the microgap between the abutment and crown. In cases with a thin soft tissue phenotype, the use of a short abutment may result in insufficient vertical mucosal thickness, potentially hindering the maintenance of the proper dimensions for supracrestal tissue adhesion. Consequently, marginal bone loss may occur as an attempt to restore the necessary vertical space. In contrast, when a short abutment is used in patients with a thick soft tissue phenotype, the crown‐abutment microgap and associated inflammatory infiltrate are positioned closer to the bone crest, possibly contributing to increased bone resorption.

Finally, an emergence profile angle of the crown > 30° showed a significant positive correlation with PBR (T1–T2) in bone‐level implants, but not in tissue‐level implants. This aligns with findings from a previous cross‐sectional study, which showed that a shallower emergence angle at interproximal sites may lower the risk of peri‐implantitis for bone‐level implants. In contrast, for tissue‐level implants, the emergence angle does not appear to be linked to an increased prevalence of peri‐implantitis [47]. The influence of prosthetic restoration emergence profile characteristics on PBR is indeed a highly debated topic. A recent meta‐analysis on this subject, which includes only four studies, suggests that implant‐supported crowns with an emergence angle ≤ 30° are associated with less PBR compared to crowns with an emergence angle > 30° [81]. However, the authors emphasize that this finding should be interpreted with caution, as the evidence supporting the study's conclusions is limited.

### 4.3 Study Limitations and Future Research

The present study presents several limitations that should be considered. The follow‐up period was limited to 1 year, which, while adequate for observing physiological bone remodeling, may not capture long‐term differences in implant performance. Furthermore, although the sample size was statistically sufficient, it was relatively small. Larger studies with extended follow‐up periods are needed to validate these findings and examine other potential factors, such as the impact of occlusal loading patterns and prosthetic design over time.

Moreover, the bone‐level implants were placed 1 mm subcrestally, following the manufacturer's recommendations. The optimal placement of the implant platform—whether at the crestal or sub‐crestal level—remains a topic of debate, with clinical studies and meta‐analyses showing conflicting results [74, 82, 83, 84]. Therefore, the differences in PBR observed in the present study may also be influenced by the subcrestal positioning of the implants.

Future research should also explore the long‐term clinical outcomes of tissue‐level versus bone‐level short implants across different anatomical settings and patient populations to better generalize the results of this investigation.

## 5 Conclusion

In conclusion, this study provides evidence that both tissue‐level and bone‐level short implants are effective options for implant‐supported rehabilitation in the posterior maxilla, with high survival rates and excellent marginal bone stability after 1 year of loading. The choice of implant type should be tailored to the individual patient's clinical scenario, with careful consideration of mucosal thickness and other local factors to optimize peri‐implant bone stability and long‐term success.

## Conflicts of Interest

The authors declare no conflicts of interest.

## Data Availability

The data that support the findings of this study are available from the corresponding author upon reasonable request.
